# Identification of pathological subtypes of early lung adenocarcinoma based on artificial intelligence parameters and CT signs

**DOI:** 10.1042/BSR20212416

**Published:** 2022-01-18

**Authors:** Weiyuan Fang, Guorui Zhang, Yali Yu, Hongjie Chen, Hong Liu

**Affiliations:** Department of Respiratory and Critical Care Medicine, The First Affiliated Hospital of Zhengzhou University, Zhengzhou 450052, Henan, China

**Keywords:** Artificial intelligence, Computed tomography, Ground-glass nodules, Lung cancer

## Abstract

**Objective:** To explore the value of quantitative parameters of artificial intelligence (AI) and computed tomography (CT) signs in identifying pathological subtypes of lung adenocarcinoma appearing as ground-glass nodules (GGNs). **Methods:** CT images of 224 GGNs from 210 individuals were collected retrospectively and classified into atypical adenomatous hyperplasia (AAH)/adenocarcinoma* in situ* (AIS), minimally invasive adenocarcinoma (MIA), and invasive adenocarcinoma (IAC) groups. AI was used to identify GGNs and to obtain quantitative parameters, and CT signs were recognized manually. The mixed predictive model based on logistic multivariate regression was built and evaluated. **Results:** Of the 224 GGNs, 55, 93, and 76 were AAH/AIS, MIA, and IAC, respectively. In terms of AI parameters, from AAH/AIS to MIA, and IAC, there was a gradual increase in two-dimensional mean diameter, three-dimensional mean diameter, mean CT value, maximum CT value, and volume of GGNs (all *P*<0.0001). Except for the CT signs of the location, and the tumor–lung interface, there were significant differences among the three groups in the density, shape, vacuolar signs, air bronchogram, lobulation, spiculation, pleural indentation, and vascular convergence signs (all *P*<0.05). The areas under the curve (AUC) of predictive model 1 for identifying the AAH/AIS and MIA and model 2 for identifying MIA and IAC were 0.779 and 0.918, respectively, which were greater than the quantitative parameters independently (all *P*<0.05). **Conclusion:** AI parameters are valuable for identifying subtypes of early lung adenocarcinoma and have improved diagnostic efficacy when combined with CT signs.

## Introduction

Lung cancer is the second most common cancer and remains the leading cause of cancer deaths for both men and women, with an estimated 1.8 million deaths (18%) [1]. Lung adenocarcinoma is the most common subtype of non-small cell lung cancer (NSCLC) and is subdivided into adenomatous precursor lesions, such as atypical adenomatous hyperplasia (AAH) and adenocarcinoma *in* *situ* (AIS), minimally invasive adenocarcinoma (MIA), and invasive adenocarcinoma (IAC), according to the recent classification of lung tumors in 2021 [2]. Ground-glass nodules (GGNs), also called subsolid nodules, are a typical imaging manifestation and can be found at any stage of lung adenocarcinoma [3, 4]. There are two types of GGNs: mixed ground-glass nodules (mGGNs) and pure ground-glass nodules (pGGNs). The detection rate of GGNs has grown dramatically as a result of the widespread use of high-resolution computed tomography (HRCT) in early lung cancer screening [5].

The management principles, surgical approach, and prognosis of GGNs differ depending on the pathological subtype, and accurate preoperative prediction of pathological subtypes is a critical step in optimizing patient management [6–8]. With adequate surgical resection, the 5-year survival rate can be as high as 100% for patients with AAH/AIS, and MIA, but MIA has the ability to transfer, while in IAC, the 5-year disease-free survival rate is 70–90% [9–11]. Therefore, it is vital to identify IAC earlier to achieve a better outcome. However, due to the heterogeneity of tumor tissue, pathological diagnosis of preoperative puncture or intraoperative rapid frozen specimens of GGNs typically differs from postoperative complete histological findings [12, 13], making accurate preoperative prediction of probable pathological subtypes difficult.

GGNs are characterized by their small size, poor contrast and highly variable shape, and most patients with GGNs lesions are asymptomatic. Some investigators reported that qualitative computed tomography (CT) findings such as the lobulation sign, irregular shape, lobulation, pleural indentation, spiculation, and the air bronchogram sign vary among early lung adenocarcinoma with GGNs [14–16]. However, manually interpreting hundreds of CT pictures of GGNs is a tremendous workload for clinicians and radiologists. Based on certain algorithms, artificial intelligence (AI) can complete a reading of HRCT in an average of 10 s and can automatically identify the location of the lesion [17]. Radiologists showed better performance when accompanied by AI software (improvement from 65.1 to 70.3%) for the detection of malignant pulmonary nodules on chest radiographs [18]. In addition, many quantitative parameters can be derived from AI to assist physicians in diagnosis and treatment, such as size and volume. Prior AI-based studies mainly concentrated on distinguishing between benign and malignant lung nodules [19–21]. It is reported that the area under the curve (AUC) of an AI-assisted CT diagnostic technique to classify lung nodules as benign or malignant was 0.95 [22]. However, only a few studies have distinguished the pathological subtypes of lung adenocarcinoma.

The purpose of the present study was to investigate the differences between AI quantitative parameters based on machine learning and deep convolutional neural networks and CT signs of GGNs with different pathological subtypes and assess the diagnostic value of a model combining the above indicators to identify pathological subtypes of lung adenocarcinoma.

## Materials and methods

### Patients

The clinical data and CT images of 210 patients with GGNs between August 2019 and June 2021 were reviewed retrospectively. Patients were eligible for inclusion if they met the following criteria: (1) the availability of a chest CT conducted to identify GGNs within 1 month before surgery; (2) the maximum diameter of GGNs was ≤30 mm; (3) the availability of complete chest CT images with a reconstructed thin-layer sequence and a layer thickness of ≤1.5 mm; (4) postoperative pathology revealed primary lung adenocarcinoma. The exclusion criteria were as follows: (1) no thin-layer sequence or slice thickness > 1.5 mm, or there were artifacts that prevented image diagnosis; (2) malignant tumor history; (3) auxiliary investigation revealed distant metastases; (4) biopsy or anti-tumor treatment prior to the CT scan.

All GGNs were divided into three groups according to pathological diagnosis: the AAH/MIS group, the MIA group, and the IAC group. The patients consisted of 146 women and 64 men with a mean age of (51.42 ± 11.15) years (range, 17–77 years). A total of 224 pulmonary GGNs included 55 AAHs/AISs, 93 MIAs, and 76 IACs. According to the medical history records, nodules were discovered in 151 cases by chest CT conducted during a physical examination, 38 cases for respiratory symptoms, and 21 cases for other reasons (digestive symptoms and others).

### Chest CT scan

A chest CT scan (supine position, ranging from the apex to the base of the lung) without contrast media was performed with a 16 or 64-detector CT system (Revolution CT, GE Healthcare, or SOMATOM Force CT, SIEMENS Healthineers) using the following scan parameters: tube voltage of 120 kV, tube current of 50–200 mA, rotation time was 0.5–1.0 s, a pitch of 1.0–1.5, conventional layer thickness of 5.0 mm, reconstruction layer thickness of 1.0–1.5 mm. All CT images were reviewed with the lung window (window width, 1500 HU; window level, −500 HU) and the mediastinal window (window width, 350 HU; window level, 40 HU).

### Pathological diagnosis

According to the World Health Organization (WHO) classification of lung tumors, the pathological diagnosis of lung adenocarcinoma includes subtypes of AAH, AIS, MIA, and IAC. All specimens were obtained from thoracoscopic or open chest biopsies, routinely fixed in 10% formaldehyde, paraffin-embedded, sectioned, and stained with hematoxylin and eosin. Two physicians with more than 10 years of experience in diagnostic pathology of chest diseases reviewed and provided the final pathological diagnosis.

### CT signs

Without knowing the pathological outcome, two senior diagnostic radiologists (more than 10 years of experience) browsed the images on the picture archive and communication systems workstation from a comprehensive view at transverse, coronal, and sagittal levels and recorded the CT signs of GGNs. The recorded CT signs were as follows: location (upper right lobe, middle right lobe, lower right lobe, upper left lobe, lower left lobe), density type (mGGN, pGGN), shape (round/round-like, irregular shape), margin (lobulation, spiculation), internal features (vacuolar sign, air bronchogram), adjacent structures (pleural indentation, vascular convergence), tumor–lung interface (clear, blurred). When two radiologists disagreed, they reached an agreement through consultation.

### AI quantitative parameters

CT images were saved in a standard format for medical digital imaging and communication, and patient information was extracted and imported into the SANMED Target Call Lung Nodule Analysis Platform (http://ctai.sanmedbio.com/, Version 1.3.2, SANMED Biotech Inc, Zhuhai, China), which is based on machine learning and deep convolutional neural networks.

The AI platform automatically reconstructed, segmented, and analyzed the CT images and labeled the lung nodules with the relevant parameter values. The parameters were defined as follows: (1) two-dimensional (2D) mean diameter (mm): (2D long diameter + 2D short diameter)/2, where 2D long diameter is the distance between the two farthest points on the largest cross-section, 2D short diameter is the shortest diameter perpendicular to the longest diameter; (2) three-dimensional (3D) mean diameter (mm): (3D long diameter + 3D short diameter)/2, where 3D longest diameter is the longest diameter of an ellipsoid with the same volume as the nodule and the standard diameter of a sphere with the same volume as the nodule is 3D short diameter; (3) volume (mm^3^): number of pixels × volume of each pixel, where the nodule consists of multiple pixels after AI segmentation, the volume of each pixel = dx × dy × dz; (4) mean CT value (HU): each pixel has a density value (HU). The mean of all the pixel density values of the nodule is the mean CT value; (5) maximum CT value (HU): the 95th percentile density value.

### Statistical analysis

SPSS 26.0 (IBM Cor P, NY, U.S.A.) and Medcalc 19.8 (Ostend Ltd, Belgium) were used for statistical analysis. Quantitative parameters were expressed as mean ± standard deviation, and comparisons between the three groups were performed using the *F* test or *Kruskal–Wallis* test (nonparametric test). Count data were expressed as frequencies (percentages), and the *Pearson χ*^2^ test or *Fisher’s* exact test (expected frequency < 5) were used for comparison among the three groups. Pairwise comparisons were made by the *Bonferroni* method with an adjustment for test levels. We selected variables with significant differences as independent variables between the two groups. *Logistic regression* (stepwise regression method) was applied to build the model and predicted probability values were obtained. The *χ*^2^ test was used to test the significance of the model and obtain regression coefficients. The goodness-of-fit for the model was determined using the *Hosmer–Lemeshow* test. A receiver operating characteristic (ROC) curve was elaborated, and the AUC, sensitivity (SE), specificity (SP), and critical value of each variable were calculated. The diagnostic accuracy was considered low when AUC was 0.5–0.7, medium when AUC was 0.7–0.9, and high when AUC was above 0.9. The AUC difference of each variable is examined by the *Delong* test. A *P-*value <0.05 was considered statistically significant.

## Results

### Demographic data

The mean age of the AAH/AIS, MIA, and IAC groups was (49.11 ± 10.28), (51.02 ± 11.21), and (53.61 ± 11.04) years, respectively, and the difference in age distribution among the three groups was statistically significant (*H*=8.419, *P*=0.004). However, a two-by-two comparison showed no statistically significant differences in age between the AAH/AIS and MIA groups, the MIA and IAC groups (both *P*>0.05). Sex, smoking history, and family history of lung cancer showed no statistical differences among the three groups (for all, *P*>0.05) (Table 1).

**Table 1:** Demographic data of AAH/AIS, MIA, and IAC groups

Variables	AAH/AIS (*n*=55)	MIA (*n*=93)	IAC (*n*=76)	*H/χ^2^*	*P*-value
Age (years)				8.419	0.004
Mean ± SD	49.11 ± 10.28	51.02 ± 11.21	53.61 ± 11.04		
Sex				1.041	0.594
Male	19 (34.55%)	25 (26.88%)	24 (34.29%)		
Female	36 (65.45%)	68 (73.12%)	52 (65.71%)		
Smoking history				0.997	0.607
Yes	4 (7.27%)	6(6.45%)	8 (10.52%)		
No	51 (92.73%)	87 (93.55%)	68 (89.47%)		
Family history of lung cancer				0.344	0.842
Yes	7 (12.73%)	15 (16.13%)	12 (15.79%)		
No	48 (87.27%)	78 (83.87%)	64 (84.21%)		

Abbreviation: SD, standard deviation.

### AI quantitative parameter analysis of GGNs

All GGNs were automatically identified and labeled by the AI platform based on machine learning and deep convolutional neural network. At the same time, quantitative parameters including 2D mean diameter, 3D mean diameter, mean CT value, maximum CT value, and volume were calculated. Table 2 shows that the differences in 2D mean diameter, 3D mean diameter, mean CT value, maximum CT value, and volume were statistically significant across the AAH/AIS, MIA, and IAC groups and between each paired comparison (all *P*<0.001). With the increasing degree of infiltration, the mean diameter, volume, the mean, and maximum density increased gradually.

**Table 2:** Analysis and comparison of AI quantitative parameters among different pathological subtypes (mean ± standard deviation)

Parameters	AAH/AIS	MIA	IAC	*H/χ* ^2^	*P*-value
2D mean diameter (mm)	8.54 ± 2.23	10.19 ± 2.68	14.98 ± 4.06	92.735	<0.001
3D mean diameter (mm)	8.44 ± 2.03	10.00 ± 2.39	14.60 ± 3.82	98.851	<0.001
Mean CT value (HU)	−631.75 ± 54.38	−594.03 ± 63.87	−516.42 ± 99.32	57.795	<0.001
Maximum CT value (HU)	−492.69 ± 135.90	−405.39 ± 152.22	−263.64 ± 169.65	37.344	<0.001
Volume (mm^3^)	352.56 ± 257.92	588.39 ± 435.76	1807.72 ± 434.42	97.325	0.001

The pairwise comparison showed that the difference in quantitative parameters between two groups was statistically significant (for all, *P*<0.05 after adjustment by the *Bonferroni* method).

### CT sign analysis of GGNs

Table 3 shows the findings of the CT signs among the three groups. The differences in the location and the tumor–lung interface (clear/blurred) were not statistically significant among the three groups (for all, *P*>0.05), yet the remaining CT signs were significantly different (for all, *P*<0.05). Each paired comparison showed that density type, shape, and lobulation between the AAH/AIS and MIA groups were statistically significant (for all, *P*<0.05), while the differences in density type, shape, lobulation, spiculation, air bronchogram sign, and pleural indentation were significant between the MIA and IAC groups (for all, *P*<0.001). The frequency of positive signs, including density type (mGGNs), lobulation and shape (irregular), gradually increased from AAH/AIS to MIA and IAC.

**Table 3:** Analysis and comparison of CT signs among different pathological subtypes

CT signs	AAH/AIS (*n*=55)	MIA (*n*=93)	IAC (*n*=76)	*χ* ^2^	*P*-value
Density type				33.199	0.001
pGGNs	47 (85.45%)^1^	61 (65.59%)^2^	28 (36.84%)^3^		
mGGNs	8 (14.55%)^1^	32 (34.41%)^2^	48 (63.16%)^3^		
Shape				23.156	0.001
Round/oval	40 (72.73%)^1^	43 (46.24%)^2^	23 (30.26%)^3^		
Irregular	15 (27.27%)^1^	50 (54.76%)^2^	53 (69.74%)^3^		
Location				8.653	0.371
Superior lobe of right lung	26 (47.27%)	31 (33.70%)	31 (36.56%)		
Middle lobe of right lung	5 (9.09%)	11 (11.96%)	11 (11.83%)		
Inferior lobe of right lung	7 (12.73%)	14 (15.22%)	13 (13.98%)		
Superior lobe of left lung	12 (21.82%)	23 (25.00%)	23 (24.73%)		
Inferior lobe of left lung	5 (9.09%)	13 (14.13%)	12 (12.90%)		
Peripheral signs					
Lobulation	20 (36.36%)^1^	50 (53.76%)^2^	71 (93.42%)^3^	50.287	0.001
Spiculation	11 (20.00%)	32 (34.41%)^2^	51 (67.11%)^3^	32.799	0.001
Internal signs					
Vacuolar sign	12 (21.80%)	28 (30.10%)	32 (42.10%)^3^	6.323	0.042
Air bronchogram	11 (20.00%)	27 (29.03%)^2^	39 (51.32%)^3^	15.884	0.001
Adjacent structure					
Pleural indentation	2 (3.64%)	12 (12.90%)^2^	35 (46.05%)^3^	41.082	0.001
Vascular convergence	39 (70.91%)	76 (81.72%)^2^	71 (93.42%)^3^	11.675	0.003
Tumor–lung interface				0.351	0.839
Blurred	33 (60.00%)	58 (62.37%)	44 (57.89%)		
Clear	22 (40.00%)	35 (37.63%)	32 (42.11%)		

^1^Indicates a statistically significant difference between AAH/AIS and MIA. ^2^Indicates a statistically significant difference between MIA and IAC. ^3^Indicates a statistically significant difference between IAC and AAH/AIS. *Bonferroni* method was used for pairwise comparison(for all, *P*<0.05 after adjustment).

**Table 4:** Results of Multivariate logistic regression analysis

Variable	β	S.E	Wald	OR	*P*-value
AAH/AIS and MIA					
3D mean diameter (X_2_)	0.322	0.093	12.078	1.381	0.001
Mean CT value (X_3_)	0.010	0.004	8.498	1.010	0.004
Irregular Shape (X_5_)	0.878	0.399	4.832	2.405	0.028
MIA and IAC groups					
3D mean diameter (X_2_)	0.544	0.107	26.036	1.722	0.001
Mean CT value (X_3_)	0.014	0.003	16.953	1.014	0.001
Lobulation (X_8_)	1.982	0.652	9.250	0.138	0.002

Abbreviations: OR, odds ratio; S.E, standard error.

### Establishment of the multivariate logistic regression model and ROC curve analysis

The variables that were statistically significant in the univariate analysis comparing the AAH/AIS and MIA groups were included in the multivariate logistic regression analysis by stepwise regression. The results showed that the mean 3D diameter (X_2_), mean CT value (X_3_), and shape (X_5_) were independent predictors for identifying AAH/AIS and MIA (Table 4), and the regression equation for model 1 was:

$$\text{Logit}(P)=3.478+0.322{\text{X}}_{2}+0.01{\text{X}}_{3}+0.878{\text{X}}_{5}$$

**Figure 1:**
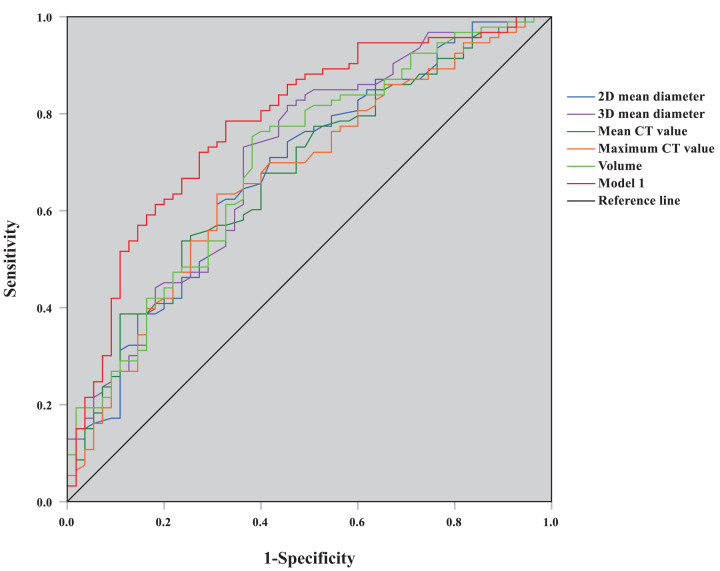
ROC curve graph of predictive model 1 and quantitative parameters for distinguishing AAH/AIS and MIA

The model 1 was statistically significant (likelihood ratio *χ*^2^ = 34.70, *P*<0.001), and all the regression coefficients also were significantly different (for all, *P*<0.05), while the goodness-of-fit for model was excellent (*χ*^2^ = 3.052, *P*=0.931). ROC curves for each quantitative parameter and predictive model 1 identifying AAH/AIS and MIA were plotted (Figure 1), and the AUCs of 2D mean diameter, 3D mean diameter, mean CT value, maximum CT value, volume, and predictive model 1 used to diagnose MIA were 0.683, 0.705, 0.676, 0.669, 0.699, and 0.779 (Table 5). Model 1, with moderate accuracy, has a higher AUC than each quantitative parameter (Supplementary Table S1, *Delong* test, for all, *P*<0.05); the threshold value of prediction probability was 0.581.

**Table 5:** Results of the ROC curve analysis

Parameters	AUC	95% CI	*P*	SE	SP	YI	Threshold
AAH/AIS and MIA							
2D mean diameter (mm)	0.683	0.594–0.772	0.001	0.613	0.691	0.304	8.98
3D mean diameter (mm)	0.705	0.617–0.793	0.001	0.731	0.636	0.367	8.33
Mean CT value (HU)	0.676	0.588–0.765	0.001	0.538	0.664	0.202	−607.00
Maximum CT value (HU)	0.669	0.579–0.759	0.001	0.634	0.691	0.325	−450.50
Volume (mm^3^)	0.699	0.611–0.786	0.001	0.753	0.618	0.371	291.00
Predicted probability 1	0.779^*^	0.701–0.857	0.001	0.785	0.673	0.458	0.581
MIA and IAC							
2D mean diameter (mm)	0.838	0.779–0.897	0.001	0.921	0.619	0.540	10.33
3D mean diameter (mm)	0.851	0.795–0.907	0.001	0.908	0.624	0.532	10.38
Mean CT value (HU)	0.738	0.662–0.813	0.001	0.566	0.806	0.372	−542.50
Maximum CT value (HU)	0.731	0.655–0.807	0.001	0.632	0.731	0.363	−325.00
Volume (mm^3^)	0.845	0.788–0.903	0.001	0.908	0.624	0.532	549.00
Predicted probability 2	0.918^**^	0.879–0.957	0.001	0.908	0.774	0.682	0.680

Abbreviations: CI, confidence interval; YI, Youden index. Compared with other quantitative parameters, ^*^P<0.05, ^**^P<0.001.

Similarly, the results of the multivariate logistic regression analysis between MIA and IAC are shown in Table 4. Model 2 consisted of the mean 3D diameter (X_2_), mean CT value (X_3_), and lobulation (X_8_). The regression equation for model 2 was as follows:

$$\text{Logit}(P)=1.744+0.544{\text{X}}_{2}+0.014{\text{X}}_{3}+1.982{\text{X}}_{8}(1)$$

**Figure 2:**
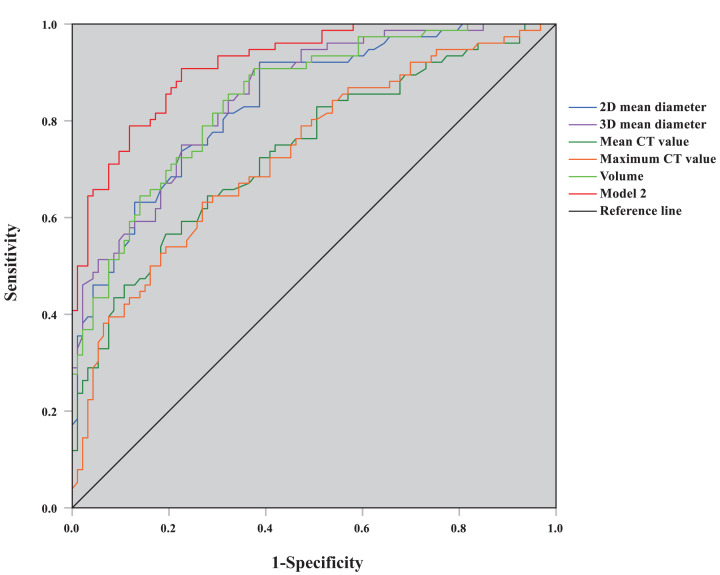
ROC curve graph of predictive model 2 and quantitative parameters for distinguishing MIA and IAC

Model 2 was statistically significant (likelihood ratio *χ*^2^ = 112.92, *P*<0.001), and all the regression coefficients also were significantly different (for all, *P*<0.05), and the goodness-of-fit for model 2 was satisfactory (*χ*^2^ = 5.206, *P*=0.735). ROC curves for each quantitative parameter and predictive model 2 identifying MIA and IAC were plotted (Figure 2), and the AUCs of 2D mean diameter, 3D mean diameter, mean CT value, maximum CT value, volume, and predictive model 2 were 0.851, 0.738, 0.731, 0.845, 0.918 (Table 5). Model 2, with high accuracy, has a greater AUC than each quantitative parameter (Supplementary Table S2, *Delong* test; for all, *P*<0.05); the threshold value of prediction probability was 0.680.

## Discussion

GGNs are a particular imaging feature that emerges as a result of local alveolar cavity infiltration. It can be a benign lesion, such as a fungal infection and bleeding, or a malignant lesion, such as AIS, MIA, or IAC [23]. Various lung adenocarcinoma subtypes require different surgical approaches and achieve different clinical outcomes. Because there is no evidence of local lymph node metastases in AAH/AIS, sublobar resection or even local wedge resection can be used for AAH/AIS instead of regional lymph node dissection. With the possibility of local lymph node metastases, MIA is also resected in the same way as AIS. However, lobectomy and regional lymph node dissection are indicated for IAC [6, 24]. Because CT images consist of hundreds of layers, clinicians and radiologists spend a copious amount of time and effort on identifying GGNs and estimating the risk of invasion. In terms of item position and classification, AI, based on deep learning outperforms manual observation, requires less manhours and decreases measurement error [25, 26], while improving accuracy and repeatability, which has been shown to exceed the traditional model in detecting benign and malignant lung nodules [27]. Identifying the pathological subtypes of GGNs can contribute to the development of a scientifically acceptable follow-up and diagnosis strategy for patients, along with an improved prognosis. In the present study, we retrospectively reviewed the data of patients with 224 GGNs to explore the value of AI quantitative parameters combined with CT signs in the differential diagnosis of pathological subtypes of lung adenocarcinoma.

The invasiveness of GGNs is closely associated with the size and density of lung nodules. Meng et al. (2021) [28] found that 2D long diameter, 2D short diameter, volume, and mean CT value could be predictive indicators of the differential diagnosis of lung adenocarcinoma, which was similar to prior studies [29–31]. In our study, the 2D mean diameter, 3D mean diameter, mean CT value, maximum CT value, and volume measured by AI were significantly different among the AAH/AIS, MIA, and IAC groups (all *P*<0.001), which were basically consistent with the mentioned studies above. The 2D mean diameter, 3D mean diameter, mean CT value, maximum CT value, and volume of GGNs were gradually increased from AAH/AIS to MIA and IAC. Yang et al. (2020) [32] found that the cut-off values for 2D mean diameter and mean CT value to identify IAC from non-IAC were 10.09 mm and −582.28 HU, respectively. Another study [30] suggested that the maximum CT value with a threshold of −300 HU can be used as an independent predictor to distinguish between pre-invasive and invasive lesions for lung adenocarcinoma. Another study indicated that the threshold of 2D mean diameter for identifying IAC was 8.12 mm. In our study, the cut-off values for the 2D mean diameter, 3D mean diameter, mean CT value, maximum CT value, and volume to identify AAH/AIS and MIA were 8.98 mm (AUC = 0.683), 8.33 mm (AUC = 0.705), −607 HU (AUC = 0.676), −450.50 HU (AUC = 0.669), and 291 mm^3^(AUC = 0.699), respectively, while the cut-off values to distinguish between MIA and IAC were 10.33 mm (AUC = 0.838), 10.38 mm (AUC = 0.851), −542.50 HU (AUC = 0.738), −325 HU (AUC = 0.731), and 549 mm^3^ (AUC = 0.845). These thresholds were slightly different from previous studies, which might be due to differences in measuring techniques, grouping, or other factors. The Fleischner Society [33] suggested that the mean diameter of lung nodules could be measured to assess changes of the size. In terms of CT value, the overall CT value and mean CT value can be used to identify the subtypes of adenocarcinoma, but standards should be unified before application. In our study, multivariate logistic regression analysis showed that the 3D mean diameter and mean CT value (both *P*<0.05) might be more suitable as indicators of size and density for the identification of pathological subtypes of GGNs.

There are different views on the role of CT signs in differentiating pathological subtypes of lung adenocarcinoma. Hsu et al. (2021) [29] reported there were significant differences between the IAC and the non-IAC with respect to lobulation, air cavity except for location, shape, interface, margin, spiculation, vessel relationship, and pleural retraction. Zhan et al. (2019) [16] found that the IAC groups had a higher frequency of mixed GGNs, bubble-like appearance, spiculation, pleural indentation, different locations, and a lower frequency of clear tumor–lung interface when compared with the AIS-MIA group. Gao et al. (2019) [14] analyzed GGNs with a diameter of ≤10 mm and the results showed that there was no significant difference in burr sign, lobulation, or pleural indentation between the IAC/MIA and AAH/AIS groups. Our study further compared the CT signs of AAH/AIS and MIA, MIA and IAC. The results showed that except for the location (*χ*^2^ = 8.653, *P*=0.371), and the tumor–lung interface (*χ*^2^ = 0.351, *P*=0.839), there were significant differences among the three groups in terms of the density type, shape, vacuolar sign, air bronchogram, lobulation, spiculation, pleural indentation, and vascular convergence sign. Further pairwise comparisons indicated that compared with AAH/AIS group, MIA had a higher frequency of mGGNs, irregular shape, lobulation, and mixed GGNs, while irregular shape, air bronchogram, lobulation, spiculation, pleural indentation, and vascular convergence sign were reported to be more common in the IAC groups compared with the MIA group. The differences between studies might be attributable to the natural processes of GGNs. AAH/AIS and MIA are still in the early stages, with a small offensive force, so there were no notable differences in CT signs between the two groups. Of course, the differences may also be linked to the technology in detecting lung nodules and the individual factors. Tumor cells actively reproduce and show aggressive invasion when GGNs develop to the IAC stage. Due to the differentiation of tumor margin cells, tumors present different growth rates or contraction of fibrous tissues within the tumor, and thus, lesions may grow with glandular, papillary, squamous, or solid patterns, resulting in the occurrence of irregular shapes, lobulation, mixed ground-glass opacity, higher frequency pleural indentation, spiculation, air bronchograms, and an abnormal dilation and distortion of blood vessels. CT signs may play a crucial role in differentiating pathological subtypes of lung adenocarcinoma. However, a meta-analysis consisting of 12 studies [34] found that CT features alone were unable to discriminate pre-invasive lesions from invasive lesions in GGNs, necessitating the development of a diagnostic mathematical model integrating CT imaging features.

In our study, multivariate logistic regression analysis was applied to variables with statistical significance in univariate analysis. The results showed that the 3D mean diameter, mean CT value, and irregular shape were independent predictive factors identifying AAH/AIS and MIA groups, and the AUC of predictive model 1 was 0.779, which was higher than the independent diagnosis of each quantitative parameter. The 3D mean diameter, mean CT value, and lobulation were independent predictive factors for the diagnosis of IAC, and the AUC of predictive model 2 with high accuracy reached 0.918, which was greater than the independent diagnosis of each quantitative parameter. Therefore, whether GGNs are in the pre-invasive or invasive stages of early lung adenocarcinoma, the diagnostic efficacy of the mixed predictive model incorporating CT signs and AI quantitative parameters was superior to that of quantitative parameters alone.

There are some limitations to the study that should be considered: (1) the study is retrospective, with some selection bias, and (2) the solid component of mGGNs was not quantified.

## Conclusions

The present study revealed that AI-based deep learning can conveniently and quickly identify GGNs. Quantitative parameters and CT signs of GGNs are different among the AAH/AIS, MIA, and IAC groups. The diagnostic efficiency of the regression model combined with the 3D mean diameter, mean CT value, irregular shape, or lobulation was higher than the single quantitative parameters.

## Supplementary Material

Supplementary Tables S1-S2

## Data Availability

To preserve patient confidentiality, the datasets generated for the present study are not publicly available but are available from the corresponding author on reasonable request.

## Abbreviations

AAH: atypical adenomatous hyperplasia
AI: artificial intelligence
AIS: adenocarcinoma *in situ*
AUC: area under the curve
CI: confidence interval
CT: computed tomography
GGN: ground-glass nodule
HRCT: high-resolution computed tomography
IAC: invasive adenocarcinoma
mGGN: mixed ground-glass nodule
MIA: minimally invasive adenocarcinoma
OR: odds ratio
pGGN: pure ground-glass nodule
ROC: receiver operating characteristic
S.E: standard error
SE: sensitivity
SP: specificity
WHO: world health organization
YI: youden index
