# Supplementary material for: Identification of pathological subtypes of early lung adenocarcinoma based on artificial intelligence parameters and CT signs
Source: Biosci Rep. 2022 Jan 18;42(1):BSR20212416. doi: 10.1042/BSR20212416 (PMC8766821; doi:10.1042/BSR20212416)
Supplement: Supplementary Tables S1-S2 [file BSR-2021-2416_supp.pdf]

Supplementary Table 1 The AUC paired analysis of predicted model 1 and quantitative parameters identifying AAH/AIS and MIA

Parameters	AUC	*Z*[*]	*P*-value
2D mean diameter (mm)	0.683	2.792	0.005
3D mean diameter (mm)	0.705	2.15	0.032
Mean CT value (HU)	0.676	2.618	0.009
Maximum CT value (HU)	0.669	2.854	0.004
Volume (mm^3)	0.699	2.37	0.018
Predicted probability1 (Model 1)	0.779		

[*]: Compared with Model 1. AAH: atypical adenomatous hyperplasia, AIS: adenocarcinoma *in situ*, MIA: minimally invasive adenocarcinoma.

Supplementary Table 2 The AUC paired analysis of predicted model 2 and quantitative parameters identifying MIA and IAC.

Parameters	AUC	Z^*	P-value
2D mean diameter (mm)	0.838	3.232	0.001
3D mean diameter (mm)	0.851	3.093	0.002
Mean CT value (HU)	0.738	4.950	< 0.001
Maximum CT value (HU)	0.731	5.169	< 0.001
Volume (mm^3)	0.845	3.159	0.001
Predicted probability 2 (Model 2)	0.918		

*: Compared with Model 2. MIA: minimally invasive adenocarcinoma, IAC: Invasive adenocarcinoma.
